# Interventional recanalization as a treatment of carotid stump syndrome caused by right internal carotid artery occlusion: A case report

**DOI:** 10.1097/MD.0000000000017152

**Published:** 2019-09-27

**Authors:** Ziqi Xu, Jinhua Wang, Benyan Luo

**Affiliations:** a Department of Neurology, The First Affiliated Hospital of College of Medicine; b Department of Neurology Beilun Branch of The First Affiliated Hospital, College of Medicine, Zhejiang University, Hangzhou, China.

**Keywords:** atherosclerosis, carotid artery occlusion, case report, endovascular therapy, ischemic stroke, stent

## Abstract

**Rationale:**

Carotid stump syndrome is a cerebral infarction caused by an embolus formed subsequent to the vortex of blood flow from the occluded stump, which then moves through the collateral vessels into the brain. The covered stent and stent-assisted coil embolization stump are the effective interventions for the carotid artery stump.

**Patient concerns:**

A 71-year-old man twice experienced left limb weakness; diffusion weighted imaging confirmed the diagnosis of cerebral infarction. Cervical computed tomography angiography, intracranial magnetic resonance angiography, and digital subtraction angiography were conducted to evaluate collateral circulation, intraluminal composition, and shape of the carotid stump.

**Diagnoses:**

The patient was diagnosed with cerebral infarction and right carotid stump syndrome.

**Intervention:**

The patient underwent interventional recanalization of the occluded internal carotid artery, which relieved his symptoms and led to satisfactory therapeutic outcomes during the clinical follow-up.

**Outcomes:**

A 9-month clinical follow-up revealed no stroke recurrence.

**Lessons:**

Interventional recanalization for the carotid artery stump syndrome is feasible. Accurate preoperative evaluation including collateral circulation, intraluminal composition, and shape of the carotid stump can assure a successful vascularization and guided management.

## 1 Introduction

Carotid atherosclerotic disease is a common etiology of ischemic strokes and accounts for about 20% cases of ischemic stroke.^[1,2]^ Previous studies have shown that the annual incidence rate of stroke is less than 3% to 5% in patients with carotid artery occlusion on drug treatments, but the stroke recurrence rate is as high as 20% in patients with poor collateral circulation.^[3,4]^ Some patients may develop recurrent cerebral embolism in the occluded internal carotid artery (ICA), namely carotid stump syndrome (CSS).^[5]^ CSS is a cerebral infarction caused by an embolus formed subsequent to the vortex of blood flow from the occluded stump, which then moves through the collateral vessels into the brain.^[5]^ The main therapy for CSS is surgery or intervention to prevent cerebral embolism.^[6–13]^ Here, we report a case of CSS that underwent interventional recanalization of the occluded ICA, which relieved the symptoms and led to satisfactory therapeutic outcomes during clinical follow-up.

## 2 Case presentation

**Figure 1:**
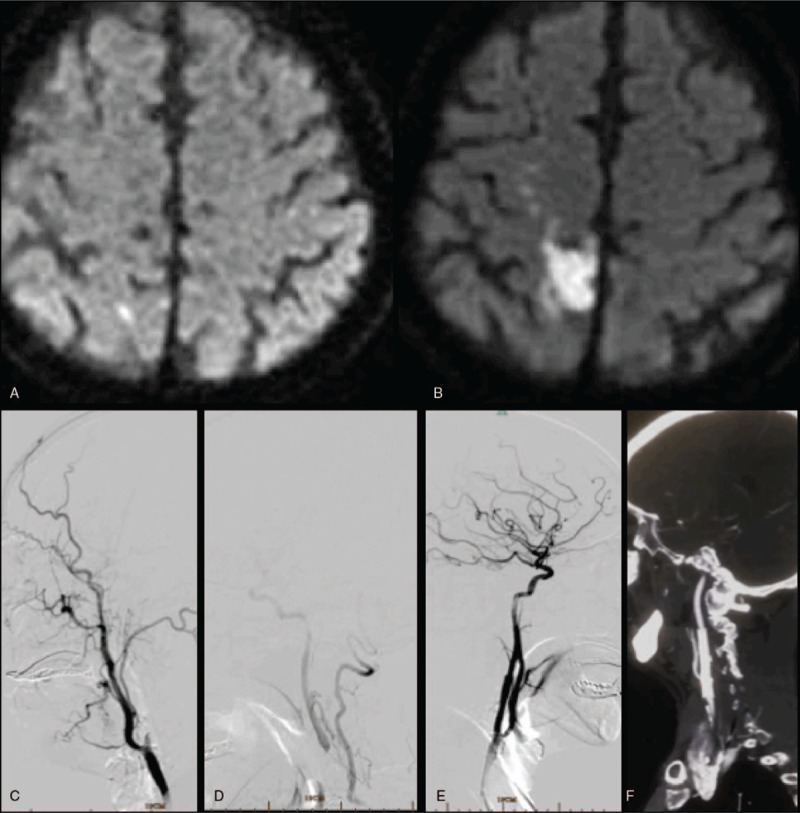
(A) Acute cerebral infarction of the right parietal cortex at the first onset; (B) acute cerebral infarction of the right parietal cortex at the second onset, spread to a larger area near the first infarct; (C and D) DSA shows occlusion of the right internal carotid artery at the start and compensation of right internal carotid artery to external carotid artery and its anastomosis branch for blood supply; (E) patent blood flow after stenting; (F) results from 3-month follow-up visits indicate patent blood flow with no restenosis in the right internal carotid artery. DSA = digital subtraction angiography.

A 71-year-old married farmer was admitted to the hospital on Jan 12, 2018 for “recurrent left limb weakness in past 7 days.” During the week before his admission, the patient suffered from weakness in the left lower limb and postural instability; however, his muscle strength of the remaining limbs was normal and his symptoms persisted for about 1 minute and then alleviated. He experienced no speech and consciousness disorders, headache, dizziness, or any other discomforts such as chest tightness and palpitation. Initially, he neither paid attention nor received any treatment for his condition. However, 2 days before admission, the patient again developed left lower limb weakness, which improved after about 1 minute. So, he visited the outpatient department and was advised a brain magnetic resonance imaging (MRI) on January 10, 2018, which showed acute infarction in the subcortical area of the right parietal lobe in diffusion weighted imaging (DWI) (see Fig. 1A). Thereafter, he was admitted to the hospital to receive further treatment. Although the patient had a previous history of hypertension, he received no standard treatment. He had a drinking history of over 40 years with an average daily drinking capacity of 250 grams of rice wine. He has also been smoking for more than 40 years with an average daily consumption of 20 cigarettes. He denied any history of diabetes and hyperlipidemia. After admission, physical examination showed alert, fluent speech, bilateral pupils of the same size and roundness, sensitivity to light reflex, normal eye movement, bilateral nasolabial groove symmetry, pharyngeal reflex, tongue protrusion in the midline, soft neck without resistance, limb muscle strength of grade 5, normal limb muscle tension, symmetrically elicited limb tendon reflex (++), negative motor coordination, negative bilateral Babinski sign, and National Institutes of Health Stroke scale (NIHSS) score of 0. Laboratory examination conducted on January 13, 2018 showed total cholesterol of 4.56 mmol/L, triglyceride of 1.65 mmol/L, high density lipoprotein cholesterol of 1.36 mmol/L, and low-density lipoprotein cholesterol of 2.63 mmol/L. The intracranial magnetic resonance angiography was normal, while carotid B model ultrasound showed right ICA occlusion. Abdominal B model ultrasound (on January 13, 2018) showed fatty liver. No obvious abnormality was detected in ultrasonic cardiogram (UCG). Upon admission, aspirin was administered to the patient as an anti-platelet therapy and rosuvastatin as a plaque stabilizing therapy. The patient refused further interventional therapy and was discharged from the hospital after drug therapy.

On April 17, 2018, the patient again experienced left lower limb weakness and postural instability. His right limb had no obvious abnormality, but his symptoms persisted and fluctuated. The physical examination done post-readmission showed body temperature of 36.7°C, pulse rate of 84 bpm, respiratory rate of 17 times/min, and blood pressure of 117/80 mm Hg. His objective neurological finding at the time of admission was normal and NIHSS score was 0. DWI (April 17, 2018) showed acute cerebral infarction in the right parietal lobe (see Fig. 1B). Upon dynamic electrocardiogram (ECG) examination, he was diagnosed with sinus rhythm, occasional atrial premature beats, occasional ventricular premature beats, and paroxysmal ST segment changes. The digital subtraction angiography results indicated right ICA occlusion and compensation for the occlusion of distal first segment (C1) of the right ICA through the anastomosis between the branches of external carotid artery and the branches of vertebral artery (see Fig. 1C and D). According to the morphological characteristics of the patient's infarct, cerebral infarction was considered as an embolization mechanism and the results of cerebral angiography suggested the occluded right ICA as the responsible blood vessel. Finally, the patient was diagnosed with cerebral infarction and right CSS. Accordingly, he was administered 100 mg aspirin combined with 75 mg antiplatelet aggregation drug clopidogrel, and 10 mg rosuvastatin to stabilize the plaque. The surgery was approved by the ethics committee of the first affiliated hospital Zhejiang University. After obtaining the informed consent from his legal relatives, percutaneous transluminal angioplasty and stenting of the occluded C1 segment of the right ICA was performed on April 25, 2018 (see Fig. 1E). Following 3 months, the carotid artery computed tomography angiography indicated patent blood flow with no restenosis (see Fig. 1F). No stroke recurrence was observed during a 9-month clinical follow-up.

## 3 Discussion

In this elderly male patient, the right ICA was occluded at the start of the right carotid artery, ischemic stroke occurred twice, and cerebral infarction foci were distributed in the right parietal cortex. In addition, the dynamic ECG and UCG results showed no abnormality, and there was no evidence of cardiogenic cerebral embolism. The patient was clinically diagnosed with CSS. Because the patient had the occlusion of the right ICA at the beginning part, the compensation of anterior and posterior communicating arteries was poor. The occluded segment of the right ICA was compensated by the anastomosis between the branches of ophthalmic, external carotid, and vertebral arteries for blood supply. Therefore, there are 2 possible causes for the embolism in this case. First, the emboli at the occluded proximal end of ICA entered into the brain through the external carotid artery and its anastomosis branches. Alternatively, the emboli at the occluded distal end of ICA entered into the brain by variations in blood flow through the ICA.

Previous studies have shown that the carotid artery occlusion accounts for about 15% of death in the patients with cerebral infarction induced by large artery stenosis.^[1]^ Also, it has been reported that the incidence rate of ipsilateral stroke is still about 5% after the carotid artery occlusion.^[14]^ Morris-Stiff et al followed up 153 patients with carotid artery occlusion using ultrasound, and found that 10.3% of the patients had spontaneous recanalization of the carotid artery occlusion and up to 23% of the patients experienced stroke in the ipsilateral occluded area.^[4]^ These conclusions were inconsistent with the study by Gomensoro et al, which states that the disease is stable and no embolism would be observed after the ICA occlusion.^[15]^ The mechanisms underlying a carotid artery occlusion-induced stroke may be as follows: first, hypodynamic cerebral infarction is caused by insufficient blood perfusion at the distal end of the occluded carotid artery. Second, cerebral infarction is caused by an embolus in the occluded aortic arch, common carotid artery, and ICA entering into the brain through the external carotid artery, Willis, and other bypass vessels.^[14]^ Omoto et al reported a case of common CSS and established the plaque movement using color Doppler ultrasound, which confirms the above mechanism.^[16]^ Therefore, there is still a high-risk of stroke recurrence after the carotid artery occlusion, and CSS cases represent the high-risk patients with internal carotid occlusion who particularly need individual intervention.

Similar to the treatment for ICA stenosis, standardized drug therapy is still the first choice for patients with ICA occlusion and can also be used in patients with CSS. Ultrasonic examination confirmed that the thrombus signal in the residual lumen changed after antiplatelet and anticoagulant therapies and the patient had no clinical onset, which directly confirmed the effect of drug therapy on CSS.^[16]^ As this patient was treated with aspirin alone at the first onset, insufficient antiplatelet strength might have resulted in stroke recurrence. Moreover, surgery and intervention are the optional strategies for CSS after internal medicine fails to work. Surgical resection of the residual lumen of the ICA and exfoliation of the tunica intima of the external carotid artery are the established surgical approaches for the treatment of CSS. Hrbáčd et al randomly divided patients with CSS into 2 groups: drug therapy group (n = 10) and surgical treatment group (n = 15), and the authors reported 1 patient in the drug therapy group with stroke recurrence, while 1 patient in the surgical treatment group died of myocardial infarction.^[7]^ Kumar et al reported that among 25 patients with CSS who underwent surgical intervention, 23 patients were relieved of symptoms. However, 1 case of facial paralysis and dysarthria, 1 case of incisional hematoma, 2 cases of cranial nerve paralysis, 1 case of visual impairment, and 1 case of death were observed.^[6]^ It can be concluded that surgical intervention to an extent is safe and effective; however, higher perioperative complications offset the clinical benefits for the patients.

The interventions for CSS are mostly demonstrated via case reports. Two surgical approaches of intervention are isolating the occluded stump of ICA with a covered or bare stent to prevent embolization,^[7–11]^ and filling the stump of the ICA with the coils supported by the stent to correct the abnormal blood flow in the residual lumen, and hence, prevent thrombosis.^[12,13]^ Due to various limitations, no guidelines have been recommended for the direct interventional recanalization of carotid artery occlusion. To our knowledge; however, this is the first reported case about interventional recanalization of the ICA occlusion in CSS.

At present, recanalization of the occluded carotid artery with interventional stenting is not fully evidenced and remains controversial.^[17]^ The application of interventional recanalization is limited by perioperative safety and distal lumen occlusion. However, relevant studies have shown that the chronic ICA occlusion, a safe and feasible condition, has a recanalization rate of 60%. The success of recanalization depends on the clinical symptoms, shape of the stump, and vascular conditions of the occluded distal landing area.^[18,19]^ According to the imaging of the ICA occlusion, Kniemeyer et al classified the ICA occlusion into 3 types (type I: subtotal stenosis with delayed, but orthograde filling of the entire ICA; type II: total occlusion of the ICA at the carotid bifurcation, but delayed orthograde filling of the cervical portion and the siphon via atypical collaterals of the proximal ICA (not always detected angiographically); type III: no visualization of the cervical ICA, but patent petrous part and siphon, due to retrograde filling of the ICA via the Circle of Willis and ophthalmic artery).^[20]^ Imaging findings showed that this patient had type II occlusion. The patient developed clinical symptoms twice within 3 months, with obvious stump, blood filling, and occlusion of the distal vascular bed at the late stage of angiography, all of which contribute towards a successful recanalization. Shojima et al performed interventional recanalization with a proximal embolic protection device, which has been proved to be effective in the treatment of chronic ICA occlusion.^[21]^ High-resolution MRI is the only approach that can display a lesion in the vascular wall in vivo consistent with the pathological results. It can provide information about the lesions on the occluded distal vessel wall and in the vascular lumen; therefore, guide the interventional recanalization and reduce the risk of intraoperative embolism.^[22]^ With the advantages of interventional techniques and surgical operation, hybrid surgery greatly improves the efficiency of recanalization and reduces the perioperative complications. It has been proved to be feasible in clinical studies.^[23,24]^

In summary, internal CSS is a rare clinical phenomenon with recurrent events in the ipsilateral carotid artery blood supply. Hypoperfusion and embolism are the key mechanisms associated with the pathogenesis of cerebral infarction. Surgery and intervention are optional strategies after an ineffective drug therapy. Therefore, interventional recanalization after an extensive evaluation can be a direct method to improve the blood flow and prevent stroke.

## Author contributions

**Conceptualization:** Ziqi Xu, Benyan Luo.

**Data curation:** Ziqi Xu, Jinhua Wang.

**Formal analysis:** Ziqi Xu.

**Funding acquisition:** Ziqi Xu.

**Investigation:** Ziqi Xu, Jinhua Wang.

**Resources:** Jinhua Wang.

**Supervision:** Benyan Luo.

**Validation:** Jinhua Wang.

**Writing – original draft:** Ziqi Xu.

**Writing – review and editing:** Ziqi Xu, Benyan Luo.
